# Vitamin B12 deficiency presenting as progressive blindness in a 33-year-old HIV-positive female patient on Efavirenz-based regimen: case report

**DOI:** 10.11604/pamj.2024.47.164.43048

**Published:** 2024-04-04

**Authors:** Gloria Lubega, Joseph Lutaakome, Moses Kibirige, Daniel Opoka, Immaculate Atukunda, Eugene Ruzagira

**Affiliations:** 1 MRC/UVRI and LSHTM Uganda Research Unit, Entebbe, Uganda,; 2 Makerere University, College of Health Sciences, Department of Ophthalmology, Kampala, Uganda,; 3 London School of Hygiene and Tropical Medicine, London, UK

**Keywords:** HIV, vitamin B12 deficiency, optic neuritis, case report

## Abstract

Optic neuritis is a rare presentation of vitamin B12 deficiency. We describe a 33-year-old female patient living with HIV presenting with progressive loss of vision for 1 week. She had a history of severe peripheral neuropathy that was managed with vitamin B12-containing tablets approximately three years before presenting with progressive loss of vision. On examination, she had no perception of light in the left eye and no perception of hand motion in the right eye. The fundus in her left eye had mild blurring of disc margins. Results from tests done showed a haemoglobin of 12.9g/dl, MCV 101fl, a serum vitamin B12 of 78pmol/l, and cytomegalovirus (CMV) test showed no active disease. She was diagnosed with optic neuritis and started on 30 mg tablets of prednisolone for 1 week with slight improvement. She was then started on vitamin B12 injections 1 mg daily for 10 days and thereafter, monthly for 6 months. She reported gradual improvement and regained her sight after 5 months treatment of with Vitamin B12 injections. Ophthalmic manifestations of vitamin B12 deficiency are not common and may present without haematological signs therefore, a high index of suspicion is required for early diagnosis and management of vitamin B12 deficiency.

## Introduction

Vitamin B12 deficiency occurs commonly among people living with HIV (PLWH) [1] and has been associated with antiretroviral therapy (ART) especially Zidovudine [2]. Vitamin B12 deficiency is complicated by various hematological and non-hematological conditions [3,4]. Life-threatening hematological conditions which include macrocytic anemia, pancytopenia, and ´pseudo´ thrombotic microangiopathy occur in up to 10% of patients with vitamin B12 deficiency [3]. The non-hematological complications include neurological disorders such as psychiatric abnormalities, myelopathy, neuropathy, subacute combined degeneration of the spinal cord, and optic neuropathy [4,5].

Optic neuropathy is an uncommon manifestation of vitamin B12 deficiency [6] and is often difficult to diagnose in resource-limited settings. Increased availability of ART significantly reduced the incidence of opportunistic infections, including cytomegalovirus (CMV) retinitis [7], which was a more common cause of blindness among PLWH in the pre-ART era. A delay in diagnosis and initiation of treatment for vitamin B12 deficiency leads to irreversible neurological disease [8]. Here in, we present a rare case of a 33-year-old HIV seropositive patient presenting with optic neuritis associated with vitamin B12 deficiency at our research clinic in Entebbe Regional Referral Hospital, Entebbe, Uganda.

## Patient and observation

**Patient information:** the patient is a 33-year-old female who first tested HIV positive in June 2012 and started ART, Tenofovir, Emtricitabine, and Efavirenz (TDF/3TC/EFV), in January 2013 with a baseline viral load (VL) of 4,868 copies/uL and a nadir CD4 count of 724 cells/mm^3^. She has a history of peripheral neuropathy in 2015 which presented as paraesthesias and non-trauma-related progressive bilateral lower limb weakness that was more pronounced on the right without associated loss of sphincter control. On examination, she required support to walk or lift her right leg, was afebrile, and did not have jaundice or lymphadenopathy. The lower limbs were non-tender with normal reflexes but had reduced power; right 4/5 and left 2/5. Results for tests done were: haemoglobin - 14g/dl, mean corpuscular volume (MCV) - 82fL (68 - 98 fL), TPHA and RPR non-reactive, HIV viral load 256 copies/ul, CD4 count 616 cells/mm^3^ and Vitamin B12 level 48.53 pmol/l. A lumbrosacral computer tomography (CT) scan is normal. She was managed with intramuscular dexamethasone 8 mg once daily for 2 days, then prednisolone 20 mg once daily for 5 days. Vitamin B complex (vitamin B1, B2, B3, B5, B6, B12) one tablet once daily for 1 month and then Neurorubine-Forte (vitamin B1, B6, and B12) one tablet once daily for 1 month. She reported a complete resolution on her next visit 3 months later. Within the same year, she was diagnosed with peptic ulcer disease and was managed with omeprazole capsules 20 mg twice daily for 2 weeks.

She was seen at the clinic every 3 months and reported no complaints on these visits until approximately 3 years later, on 31^st^ January 2018 when she presented with reduced appetite. She had a CD4 count of 547 cells/mm^3^ and undetectable VL. She was given multivitamin tablets for 3 months. On 18^th^ May 2018, she presented with reduced appetite and weight loss of 2kg since her previous visit. She was given multivitamin tablets once daily for 1 month and an albendazole tablet 400mg stat. On 20^th^ June 2018, she presented with painless progressive loss of vision for one week without other neurological symptoms. She reported reduced vision that started in the left eye and progressed to complete blindness in that eye. This was followed by reduced vision in the right eye, which had progressed to partial blindness (seeing only shadows).

**Clinical findings:** eye examination by the ophthalmologist revealed no perception of light in the left eye, and perception of hand movements in the right eye; mildly dilated pupils with sluggish reaction to light, the lenses were clear. The right eye fundus had a disc with regular margins, while the left eye fundus had mild blurring of disc margins. Normal macular seen.

**Timeline of the current episode:** 2018-06-2 presented with progressive loss of vision for one week starting in the left eye.

**Diagnostic assessments and therapeutic interventions:** tests done showed; haemoglobin of 12.9 g/dl, increased MCV of 101fL, and undetectable VL. A diagnosis of optic neuritis was made, and the patient started on prednisolone tablets 30mg once daily for 1 week and vitamin A 200,000IU stat. At a follow-up visit on the 9^th^ of July 2018, she reported slight visual improvement. Visual acuity was counting fingers at 1 meter for both eyes. Results for tests done at this visit showed; low vitamin B12 at 78pmol/l, positive serum CMV IgG at >250 AU/mL, and a negative serum CMV IgM.

**Diagnosis:** a diagnosis of optic neuritis secondary to vitamin B12 deficiency was made.

**Therapeutic interventions:** the patient was started on vitamin B12 injections 1 mg daily for 10 days and 1 mg monthly thereafter for 6 months. She did not report any adverse reactions to the vitamin B12 injections.

**Follow-up and outcome:** she reported gradual improvements on her subsequent visits, reporting normal sight on her visit on 08^th^ Nov 2018. The patient was unable to pay for a repeat vitamin B12 test, nor was she able to pay for the consultation at the ophthalmologist. She was happy to have regained her sight. She continued receiving vitamin B12 injections monthly at the clinic until 08^th^ May 2019. Likewise, she was switched to oral vitamin B complex tablets one daily for life. She requested a transfer to her village during COVID-19 restrictions due to the loss of her job. She was advised to continue vitamin B complex tablets for life because of the unavailability of vitamin B12 testing in the rural area she transferred to.

**Patient perspective:** the patient appreciated the efforts of the clinical team and was happy to regain her sight. She promised to continue taking her oral vitamin B complex tablets for life.

**Informed consent:** the participant gave the author's consent to write up this case.

## Discussion

Ophthalmic manifestations of vitamin B12 deficiency are not common [6]. The majority of the cases reported present with optic nerve atrophy, but rarely do they present with optic neuritis [6]. The exact pathophysiological mechanism by which vitamin B12 deficiency affects the optic nerve is not well understood however, it has been suggested that optic nerve damage may occur via degeneration [6]. Neurologic symptoms of vitamin B12 deficiency rarely occur without haematological signs [5]. Even though our patient had low vitamin B12 levels from 2015, her haemoglobin and MCV parameters were within normal range, with MCV increasing slightly at the point of presentation with blindness. A high level of suspicion is required to diagnose vitamin B12 deficiency in patients with atypical presentation [9].

Vitamin B12 deficiency is caused by malnutrition, malabsorption and genetic defects in the cellular delivery and uptake of vitamin B12 [4]. Dietary sources of vitamin B12 are animal products, and thus strict vegetarians are at an increased risk of developing vitamin B12 deficiency. Our patient was not a known vegetarian and therefore reported no dietary restrictions with respect to animal-derived foods. However, she had a history of Helicobacter pylori infection and treatment with protein pump inhibitors (omeprazole). Helicobacter pylori infection and long-term ingestion of proton pump inhibitors and H2 receptor antagonists can reduce the absorption of vitamin B12 [4].

Previous studies have reported a common occurrence of vitamin B12 deficiency among PLWH, however, the association between HIV and vitamin B12 deficiency is not well understood [1]. The most common causes of blindness among PLWH are opportunistic infections like CMV retinitis and neoplasms [7]. In the era of ART, the prevalence of opportunistic infections has greatly reduced. Our patient´s serum CMV test showed she had no active CMV disease. Studies report a similar efficacy between injectable vitamin B12 and oral Vitamin B12 [10]. However, depending on the cause of the vitamin B12 deficiency, injectable vitamin B12 may be required. Our patient had previously been treated with vitamin B12-containing supplements however, the treatment was given for a very short time, which may explain the progression of the disease process and eventual involvement of the optic nerve. Moreover, serum vitamin B12 levels were not monitored to establish a response to treatment. Regular monitoring of vitamin B12 levels is required among PLWH, especially for those with a history of low serum vitamin B12 levels.

## Conclusion

A high index of suspicion for vitamin B12 deficiency is required for an early diagnosis for timely and proper management of the vitamin B12 deficiency. Further research is required to determine the pathophysiological mechanisms by which vitamin B12 deficiency leads to optic neuropathy.
